# Zoledronate and SPIO dual-targeting nanoparticles loaded with ICG for photothermal therapy of breast cancer tibial metastasis

**DOI:** 10.1038/s41598-020-70659-x

**Published:** 2020-08-13

**Authors:** Zichao Jiang, Jingyi Li, Sijie Chen, Qi Guo, Zhaocheng Jing, Biying Huang, Yixiao Pan, Long Wang, Yihe Hu

**Affiliations:** 1 Department of Orthopedics, Xiangya Hospital, Central South University, Changsha, 410008 Hunan China; 2 Departmen of Ultrasound Diagnosis, The Second Xiangya Hospital, Central South University, Changsha, 410011 Hunan China

**Keywords:** Breast cancer, Breast cancer, Nanoscale materials

## Abstract

Currently, nanoparticles (NPs) for cancer photothermal therapy (PTT) have limited in vivo clearance, lack targeting ability and have unsatisfactory therapeutic efficiency. Herein, we report a dual-targeting and photothermally triggered nanotherapeutic system based on superparamagnetic iron oxide (Fe_3_O_4_) and indocyanine green (ICG)-entrapped poly-lactide-co-glycolide modified by ZOL (PLGA-ZOL) NPs (ICG/Fe_3_O_4_@PLGA-ZOL) for PTT of breast cancer tibial metastasis, which occurs frequently in the clinic and causes challenging complications in breast cancer. In this system, both ICG and Fe_3_O_4_ can convert light into heat, while NPs with Fe_3_O_4_ and ZOL can be attracted to a specific location in bone under an external magnetic field. Specifically, the dual-targeting and double photothermal agents guaranteed high accumulation in the tibia and perfect PTT efficiency. Furthermore, the in vivo studies showed that ICG/Fe_3_O_4_@PLGA-ZOL NPs have extraordinary antitumor therapeutic effects and that these NPs can be accurately located in the medullary cavity of the tibia to solve problems with deep lesions, such as breast cancer tibial metastasis, showing great potential for cancer theranostics.

## Introduction

Bone metastasis (BM) is the most frequent complication in patients with breast cancer, and the incidence of BM in breast cancer patients is up to 60–70%^1^. The rate of BM accounted for approximately 20% of over 230,000 newly diagnosed patients with invasive breast cancer and 70% in the postmortem examination of patients dying of breast cancer^2,3^. Tumor cells can remarkably destroy the balance between osteoblasts and osteoclasts in skeletal microenvironment, and the interactions among them can also secrete the cytokines to promote the growth of cancer cells, ultimately enhance osteoclast differentiation and bone resorption^4,5^. The 5-year survival for patients with BM decreases to 8.3% from 75.8% in patients without BM^6^. Currently, BM is a great challenge in clinical practice, leading to a series of skeletal related events (SRE), including pain, pathologic fracture, and spinal cord compression, reduced quality of life, and eventually develop into disability^7^. Currently, for breast cancer patients with BM, the main clinical treatment is surgery, radiotherapy, chemotherapy or analgesics. Surgery and analgesic can only control local symptoms and give limited improvement of quality of life. Chemotherapy or radiotherapy results in a series of systemic damage are still unsolved. It required a new therapy with a lower side effect, in addition, with higher efficacy.

Photothermal therapy (PTT) can be induced by near-infrared (NIR) light. A specific photothermal agent that generates heat stimulated by laser with a proper wavelength can locally heat to a high temperature (> 45 °C). In general, tumor cells are more susceptible than normal cells to temperature rising^8,9^, leading to ablation of tumor cells^10–12^. PTT has recently drawn great attention due to its high therapeutic efficacy, deep tissue penetration ability and low invasiveness compared to that of traditional chemotherapy, radiotherapy and surgery^13–15^. Various nanoscale photothermal agents, including metal NPs (gold NPs^16–18^ and copper NPs^19–21^), carbon nanotubes^22–25^ and NIR dye-based nanocarriers^26,27^, have outstanding NIR light absorption properties, and incorporating these agents into nanoparticles (NPs) for cancer PTT has been extensively researched. However, many agents have limits, which will impede their clinical applications. Metal NPs and carbon nanotubes have poor biodegradability, and many NIR dyes have unsatisfactory biocompatibility and even toxicity to living systems.

Indocyanine green (ICG) is a water-soluble tricarbocyanine dye that exhibits absorption and emission maxima in the NIR region at approximately 740 and 800 nm, respectively^28^. The only NIR agent approved by the U.S. Food and Drug Administration (FDA), ICG has been widely used in research on PTT^11,15^. Although its biocompatibility is nearly perfect, several challenges hinder the direct application of ICG in PTT, including its temperature- and light-dependent optical properties, instability in aqueous solution, and extremely high in vivo clearance with a short half-life of 2–4 min^28,29^. In addition, effective cancer treatment requires good targeting. Several studies have reported the encapsulation of ICG into appropriate nanocarriers that could enhance its stability in a biological fluid system, remarkably increasing its accumulation in cancer lesions through the enhanced permeability and retention (EPR) effect and enhancing its PTT efficiency^12,15,22,30,31^. Poly-lactide-co-glycolide (PLGA) is one of the most widely used nanocarriers due to its excellent biodegradability and biocompatibility. PLGA is also approved by the FDA, and its perfect loading capacity has been proven by many successful NPs established by previous researchers^12,14,32,33^.

In this work, we propose to overcome the intrinsic issues of ICG by encapsulating it into PLGA NPs (ICG@PLGA) and applying these NPs for PTT of BM, while simple assembly of ICG@PLGA still lacks targeting. Superparamagnetic iron oxide (Fe_3_O_4_) has low toxicity and has been widely researched in nanomaterials due to its unique magnetic properties, significant effects on T2 relaxation for magnetic resonance imaging (MRI), and excellent photo-absorbing ability^34–36^. Moreover, Fe_3_O_4_ can be employed as a sophisticated targeting moiety. For example, a magnetic field can attract Fe_3_O_4_-modified NPs to a specific location. Compared with molecular targeting, magnetic targeting relies on basic physical interactions, has good repeatability, is not limited by specific receptor expression, minimizes side effects to normal tissue, and may improve the efficacy of PTT^11,13,37,38^. Zoledronic acid (ZOL) is a representative third-generation bisphosphonate drug and has been applied clinically for the treatment of bone diseases. ZOL attenuates cancer-induced bone erosion, significantly decreases the risk of SREs and re-establishes bone density via a direct effect on the osteoclastogenesis process^39,40^. ZOL has a high affinity for hydroxyapatite and can be rapidly and selectively trapped in bone tissues after entering the systemic circulation due to its unique ‘P–C–P’ structure. Once bound to bone, ZOL will remain for several months. Thus, ZOL can also be employed as a target factor for bone. Moreover, bisphosphonate-coated PLGA NPs can improve therapeutic efficacy by targeting bone, as shown in previous studies^41–43^. However, to the best of our knowledge, a nanotherapeutic system that is based on magnetically and photothermally triggered ICG@PLGA NPs and targets against BM of breast cancer has been rarely reported.

**Figure 1:**
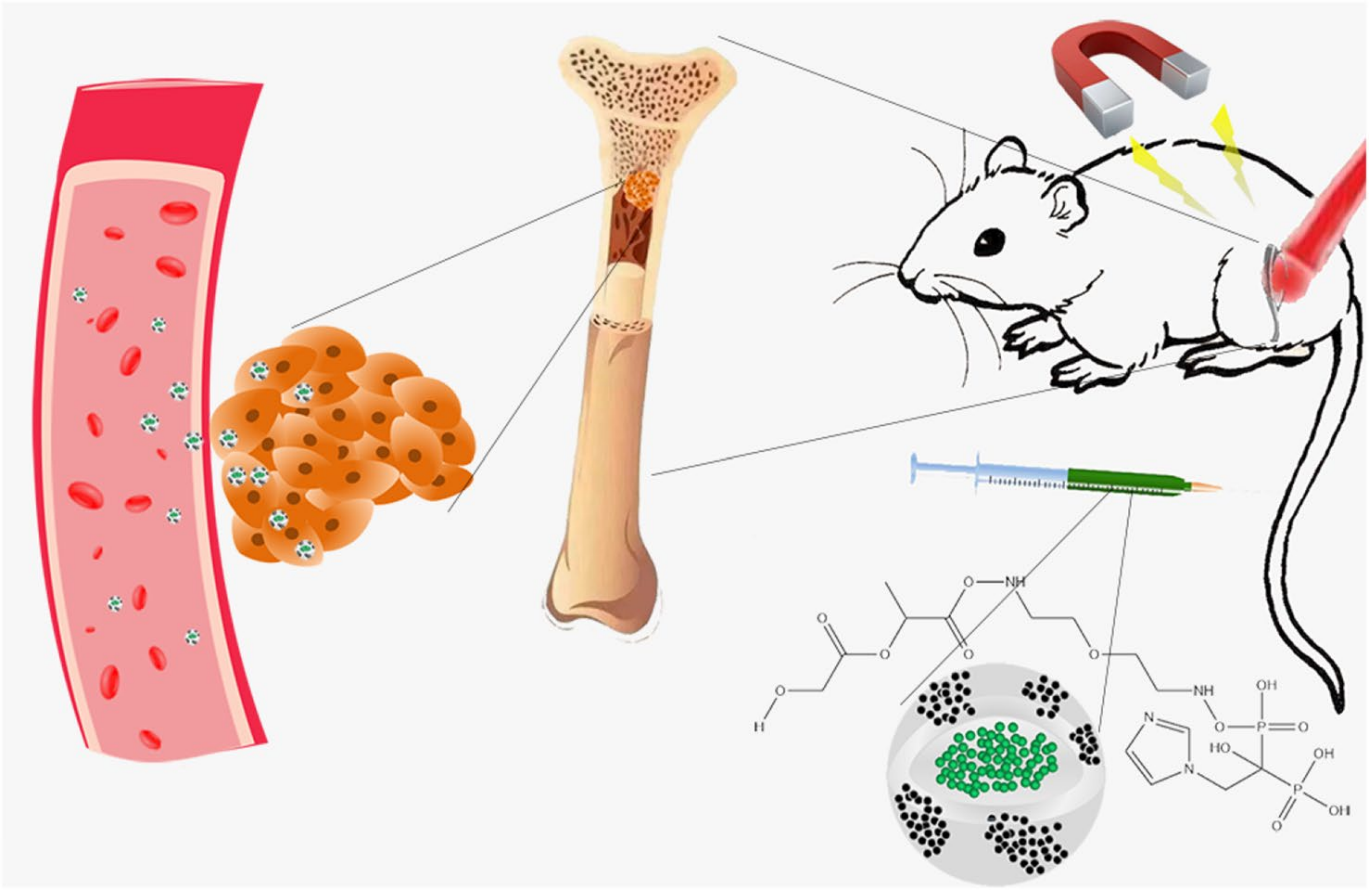
Schematic drawing by Adobe Photoshop (Verion 13.0, U.S.) showed the structure of the ICG/Fe_3_O_4_@PLGA-ZOL and therapeutic procedure of bone metastasis of breast cancer with the ICG/Fe_3_O_4_@PLGA-ZOL.

Herein, we fabricated a novel NP system, ZOL-PLGA NPs loaded with ICG and Fe_3_O_4_ (ICG/Fe_3_O_4_@PLGA-ZOL), by integrating the photothermal ability of ICG with the magnetic property of Fe_3_O_4_ and the bone affinity of ZOL to target tumor sites and display high-efficiency PTT, thus alleviating cancer-induced bone resorption in mice (Fig. 1). In addition, ZOL alone may also provide a therapeutic effect.

## Materials

ICG, PLGA (actide:glycolide = 50:50, Mw = 10,000) and polyvinyl alcohol (PVA, Mw = 30,000–70,000) were all purchased from Sigma-Aldrich (USA). The Fe_3_O_4_ NPs (diameter = 10 nm) treated with oleic acid were purchased from Ocean Nanotech Co., Ltd. (USA). ZOL (Mw = 272.09) was obtained from Energy Chemical (Shanghai, China), and NH_2_–PEG–NH_2_ was purchased from Ponsure Biotechnology (Shanghai, China). Deionized (DI) water was purified using a Milli-Q gradient system. DiI, DAPI, DiR iodide and the Calcein AM-PI double staining kit were all obtained from Beyotime Biotechnology (Changsha, China). The other reagents were of analytical grade and were used without further purification. The 4T1 mouse breast cancer cells and RAW264.7 mouse leukemia cells of monocyte macrophages were obtained from Xiangya Hospital, Central South University (China) and cultured as recommended. A circular magnet iron (diameter of 1.2 cm, with magnet field strength of 6 Gs) was used for magnetic targeting, and a hand-held magnetic field strength meter (TD8620, Tunkia Co., Ltd., Changsha, China) was used to measure the magnetic field. An H-7600 transmission electron microscope (TEM, Hitachi H-7600, Japan) was used to observe the structure of the NPs. An IVIS Spectrum imaging system (PerkinElmer, U.S.) was used for fluorescence imaging in vivo. A Viva CT-80 (SCANCO Medical AG, Zurich, Switzerland) system was used for micro-CT scanning. All experiments involving the use of animals were performed in accordance with the relevant guidelines and regulations approved by the Ethics Committee at Xiangya Hospital of Central South University in China.

### Synthesis of ZOL–PEG–PLGA conjugates

ZOL–PEG–PLGA conjugates was fabricated followed former reports^44–46^, *N*-hydroxysuccinimide (NHS) and dicyclohexylcarbodiimide (DCC) were used to activate the PLGA in dichloromethane (DCM) while being stirred at room temperature until sufficiently reacted. NH_2_–PEG–NH_2_ was added in the equivalent molar rate of PLGA, diisopropylethylamine (DIEA), triethylamine (TEA) and was purified after the reaction. The formed PLGA–PEG–NH_2_ was further conjugated with ZOL with *N*,*N′*-carbonyldiimidazole (CDI) as a conjugation linker. First, ZOL was activated by CDI before reacting with PLGA–PEG. Subsequently, 100 mg ZOL was dissolved in dimethyl formamide (DMF) in a flask, then 90 mg CDI (moisture free) and a small amount TEA were added. The whole device was tightly closed and maintained under nitrogen flow for 24 h at 60 °C, and the organic solvents were then evaporated. The precipitates were used to activate ZOL and were washed twice with acetonitrile and then dried under vacuum. Then, 23 mg of the activated ZOL was added to 1 g of PLGA–PEG, dissolved in dimethyl sulfoxide (DMSO) with TEA in a tightly closed flask under nitrogen flow and reacted for at least 12 h. The mixture was freeze dried after purification by molecular sieve and reverse chromatography. Proton nuclear magnetic resonance (^1^HNMR) spectroscopy was used to confirm the successful conjugation of ZOL–PEG–PLGA.

### Preparation of ICG/Fe_3_O_4_@PLGA-ZOL NPs

ICG/Fe_3_O_4_@PLGA-ZOL NPs was fabricated as the former report^7,14^, 100 mg ZOL–PEG–PLGA was weighed and mixed with 3 ml DCM. After stirring well, 0.5 ml of the ICG solution (suspended in DI water, 4 mg/ml), 0.2 ml of the Fe_3_O_4_ NP suspension (31 mg Fe/ml) and 15 ml of the PVA solution (4% w/v, stored at 4  °C) were added in order. Then, the samples were emulsified for 2 min with an ultrasonic processor under proper conditions (50% energy, 5 s on and 5 s off, 1 min working time). The resulting emulsion was mixed with 20–25 ml DI water and stirred in a magnetic stirrer for at least 2 h to ensure that the methylene chloride was completely evaporated. The resulting mixture was washed three times with DI water (10 000 rpm, 10 min, 4 °C). Finally, the NPs were resuspended with 10 ml DI water. All procedures above were performed in a darkroom. DiI or DiR was added before the PVA solution was poured in to fabricate the DiI- or DiR-coated NPs, and the remaining steps were the same as the above procedure.

### Characterization

The size distribution and zeta potential of the ICG/Fe_3_O_4_@PLGA-ZOL NPs were analyzed by a Malvern size and zeta potential analyzer (Malvern Nano ZS, UK) at room temperature (20 °C). The size distribution was measured again 24 h later to ensure the stability of the colloidal solution. The ultraviolet–visible–near-infrared (UV–Vis–NIR) absorption spectra of the free ICG, Fe_3_O_4_@PLGA NPs and ICG/Fe_3_O_4_@PLGA NPs (0.2 ml of each sample, equivalent ICG concentration of 6 µg/ml, PLGA concentration at 60.7 µg/ml) were collected by a steady-state spectrophotometer (BioTek Instruments, UK). The morphology of the ICG/Fe_3_O_4_@PLGA-ZOL NPs was observed by a TEM (Hitachi H-7600). A magnet was set near a cuvette filled with an ICG/Fe_3_O_4_@PLGA-ZOL NP solution to estimate the magnetic feature of the NPs. The ICG drug loading and encapsulation efficiency were obtained by a Cary 5000 UV–Vis–NIR spectrophotometer (USA) and calculated according to the following formula^47^:

$$ \begin{aligned} {\text{Drug loading }}\left( \% \right) \, = & {\text{ W}}_{{\text{E}}} /{\text{ W}}_{{\text{N}}} \times {1}00\% \\ {\text{Encapsulation efficiency }}\left( \% \right) \, = & {\text{ W}}_{{\text{E}}} /{\text{ W}}_{{\text{T}}} \times {1}00\% \\ \end{aligned} $$

where W_E_ is the amount of encapsulated ICG in the NP after centrifugation, lyophilization, and redissolution in DMSO and is measured by a UV–Vis–NIR spectrophotometer. W_N_ is the mass of the NPs after centrifugation and lyophilization, and W_T_ is the total amount of added ICG.

The amount of Fe_3_O_4_ (W_1_) in the final NP sediment was estimated based on the weight of the ICG/Fe_3_O_4_@PLGA NPs (W_2_) and ICG@PLGA NPs (W_3_): W_1_ = W_2_–W_3_.

### In vitro PTT effect

Aqueous suspensions of free ICG, ICG@PLGA NPs, PLGA–Fe_3_O_4_ NPs, ICG/Fe_3_O_4_@PLGA-ZOL NPs [1 ml of each sample in a 1.5 ml Eppendorf (EP) tube, with an equivalent ICG concentration of 18 µg/ml] and simple PLGA NPs were irradiated by an 808 nm laser at 1 W/cm^2^ for 7 min (T808F2W, Minghui Optoelectronic Technology, China)^12^. PBS and free ICG were set as the negative and positive controls, respectively. The temperature was measured in 30 s intervals by an infrared thermal imaging camera (FLIR C2, USA). The photostability of NPs was measured under 808 nm NIR laser irradiation with an ON/OFF cycle^48^. The free ICG, ICG@PLGA NPs, ICG/Fe_3_O_4_@PLGA-ZOL NPs (dissolve in 1 ml of PBS, with an equivalent ICG concentration of 18 µg/ml) was irradiated for 2 min (laser ON) and then cooled at room temperature for 1 min (laser OFF). The cycle was repeated 3 times.

### Calculation of photothermal conversion efficiency

An EP tube with 1 ml of the ICG/Fe_3_O_4_@PLGA-ZOL NP suspension (8 mg/ml) was irradiated by the laser, the NIR laser was turned off after reaching the peak temperature stage and cooled to the initial temperature, and the temperature was recorded at 15 s intervals. Following the former reports^14,49^, the photothermal conversion efficiency of ICG/Fe_3_O_4_@PLGA-ZOL NPs could be calculated by Eq. (1):

1 $$ \eta = \frac{{hS(T{\max} - Tsur) - Qdis}}{{I(1 - 10^{ - A808} )}} $$

where *h* is the heat transfer coefficient, *S* is the surface area of the container, *T*_max_ represents the equilibrium temperature of the aqueous dispersion of ICG/Fe_3_O_4_@PLGA-ZOL NPs under irradiation, *T*_*sur*_ is the ambient temperature of the environment, and *Q*_*dis*_ is the baseline energy inputted by the sample cell, which can be measured independently by irradiating the sample cell with water only. *I* expresses the laser power density, *A*_808_ is the absorbance of the NPs at 808 nm, and *hS* can be derived from the following equation:

2 $$ hS = \frac{msCs}{\tau } $$

To obtain *τ*, which represents the time constant for heat transfer from the system, a dimensionless driving force, temperature (θ), is introduced using the maximum system temperature,

3 $$ \theta = \frac{{T - T{\text{sur}}}}{T\max - Tsur} $$

4 $$ t = - \tau In(\theta ) $$

The linear regression curve of the temperature cooling time (t) vs − ln(θ) of the NPs can determine the *τ*, and m_s_ is the mass of aqueous NPs. Substituting C_s_, the heat capacity of deionized water as a solvent, into Eq. (2), Eq. (1) can be used to obtain the photothermal conversion efficiency of ICG/Fe_3_O_4_@PLGA-ZOL NPs.

### In vitro cell experiments

The biocompatibility of ICG/Fe_3_O_4_@PLGA NPs and their photothermal ablation of tumor cells in vitro were evaluated by the Cell Counting Kit (CCK-8) assays. First, the 4T1 and RAW264.7 cells were incubated in a 96-well plate with 0.5 × 10^4^ cells per well (0.1 ml medium) for 24 h in an incubator (37 °C, 5% CO_2_). The ICG/Fe_3_O_4_@PLGA NPs with different ICG concentrations (18, 9, 3.6, 1.8, 0.9, 0.36 and 0 µg/ml) were added and divided into 3 groups (n = 6): (1) magnet only, (2) laser (1 W/cm^2^, 3 min) only, and (3) magnet + laser; PBS without NPs was set as a control. Then, the cells were incubated with NPs for 12, 24 and 48 h, and the CCK-8 reagent was added. The absorbance at 450 nm of each well was measured by spectrophotometry after further incubation for 3 h to determine the cell viability.

Confocal laser scanning microscopy (CLSM, Zeiss LSM 510) was employed to estimate the cell uptake capacity of ICG/Fe_3_O_4_@PLGA NPs and apoptosis of cells after NIR irradiation. The 4T1 cells were cultured on a confocal imaging dish at a density of 1 × 10^6^/ml. After 24 h in an incubator, the cells were treated with 200 µl DiI-coated ICG/Fe_3_O_4_@PLGA NPs for 3 h in an incubator. Then, we stained the cells with DAPI and imaged them to ensure photothermal ablation of the tumor cells. The 4T1 cells were incubated in confocal imaging dishes for 24 h similar to the preceding steps, and the dish were divided into 4 groups according to different interventions (n = 3): (1) PBS without laser, (2) PBS with laser, (3) ICG/Fe_3_O_4_@PLGA NPs without laser, and (4) ICG/Fe_3_O_4_@PLGA NPs with laser. Groups 1 and 4 were irradiated with 808 nm NIR at 1 W/cm^2^ for 3 min. Finally, the cells were stained with DAPI and the Calcein AM-PI double staining kit and imaged to evaluate the cell killing efficiency.

### Animal model of BM

The BALB/c mice (female, aged 6 weeks, 20–25 g) were intra-tibially injected with the 4T1 breast cancer cells using an assay that was proven by preceding research^43,50^. First, the mice were anesthetized by intraperitoneal injection of 0.06 ml pentobarbital sodium at a w/v ratio of 2.5%. Then, 4 × 10^5^ 4T1 cells in 20 µl PBS were implanted in the medullary cavity of the right tibial. A 29 gauge insulin syringe was used for pricking the bone, while another was used for intra-tibial injection of 4T1 cells.

Ultrasound (US, S3000, Siemens, Mountain View, CA, USA) examination was performed to observe the leg of the mice from the 3rd day post-modeling to confirm that molding was successful and BM were treated in the early stage. In our experience, these changes can be mediated for successful modeling: (1) the tibial cortex in the ultrasound images is observed discontinuously or roughly (most important and representative change); (2) the maximum diameter of the modeling leg has increased > 1.0 mm compared with that of the control leg; and (3) the distance between tibial tuberosity and the skin of the modeling leg has increased > 0.2 mm compared with that of the control leg.

### Fluorescence imaging

DiR (Ex = 748 nm, Em = 780 nm) is an NIR probe with red fluorescence. To evaluate the efficacy of the bone-targeting ability of the ICG/Fe_3_O_4_@PLGA-ZOL NPs, DiR-coated NPs were used to observe the distribution of the NPs in vivo. Thirty-six modeling mice were randomly divided into 4 groups (n = 9): (1) without targeting, (2) with magnetic targeting only, (3) with ZOL targeting only, and (4) with magnetic targeting and ZOL. Then, 0.2 ml of 8.0 mg/ml DiR-coated NPs was injected intravenously via the tail. In addition, a circular magnet (10 mm in diameter and 3 mm in thickness) was fastened to the proximal tibia of all mice with glue. The mice injected with NPs without targeting were used as controls. The IVIS Spectrum imaging system (PerkinElmer, U.S.) was used to image 3 mice of each group at 12, 24 and 48 h. The region of irradiation (ROI) was analyzed by IVIS Spectrum imaging software. To identify the time at which the NP concentration in the experimental leg was maximum and to ensure the best time for NIR irradiation, the mice were sacrificed by cervical dislocation, and important organs and both legs with muscles removed were harvested and imaged.

### In vivo PTT to BM

The modeling mice were randomly divided into 6 groups (n = 4): (1) saline with NIR laser, (2) ICG/Fe_3_O_4_@PLGA-ZOL NPs without NIR laser, (3) ICG@PLGA NPs with NIR laser, (4) ICG@PLGA-ZOL NPs with NIR laser, (5) ICG/Fe_3_O_4_@PLGA NPs with NIR laser, and (6) ICG/Fe_3_O_4_@PLGA-ZOL NPs with NIR laser. A 200 µl aliquot of saline or NP solution was intravenously injected into the mice (equivalent ICG concentration of 18 µg/ml, concentration of NPs was 8.0 mg/ml). All mice were fastened with a circular magnet iron in the proximal tibia on the experimental side. Groups 1, 3, 4, 5 and 6 were irradiated in the proximal tibia with an NIR 808 nm laser at 1 W/cm^2^ for 5 min at 24 h post-injection of NPs or PBS. The temperature change was monitored by an infrared thermal camera at 30 s intervals. The treatment procedure was repeated 24 h after the first irradiation was completed. The weight and leg circumference of the proximal tibia of each mouse were recorded every day.

The mice were sacrificed by cervical dislocation at 13 days or died because of BM before that. The survival days of every mouse in the different groups were recorded. Then, the important organs (heart, liver, spleen and kidney) and the leg that was cut off from the proximal femur were harvested and fixed in 4% paraformaldehyde. The legs were observed with US detection, microcomputed tomography (micro-CT) was performed by a Viva CT-80 system, and the images were analyzed using SkyScan CT analysis software (SCANCO Medical AG, Zurich, Switzerland). The region of interest was set to 50 slides below the growth plate^51^, and the bone volume (BV) and trabecular number (Tb.N) were measured^45^. Staining was performed with hematoxylin and eosin (H&E), and the leg tissue slices were also stained using TRAP assays to evaluate the osteoclaststy in lesion.

## Results and discussion

### Characterization

**Figure 2:**
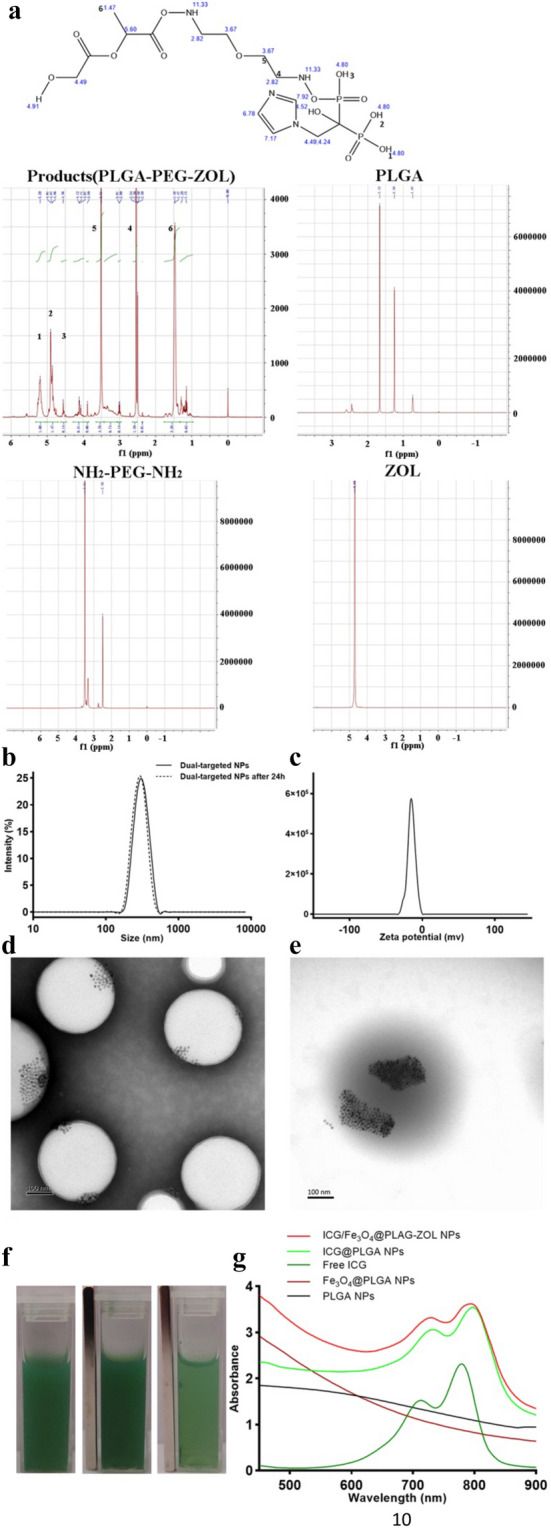
(**a**) Chemical formula and reference ^1^HNMR shift of PLGA-PEG-ZOL and final ^1^HNMR spectrum of the products (PLGA-PEG-ZOL), PLGA, NH_2_-PEG-NH_2_ and ZOL. (**b**) Size distributions of ICG/Fe_3_O_4_@PLGA-ZOL NPs at 20 °C. (**c**) Zeta potential of ICG/Fe_3_O_4_@PLGA-ZOL NPs at 20 °C. (**d**) TEM images of ICG/Fe_3_O_4_@PLGA-ZOL NPs at ×70,000, scale bar 100 nm, with negative staining. (**e**) TEM images of ICG/Fe_3_O_4_@PLGA-ZOL NPs, ×70,000, scale bar 100 nm, without negative staining. (**f**) ICG/Fe_3_O_4_@PLGA-ZOL NPs under an external magnetic field. (**g**) UV–Vis–NIR absorption spectra of the ICG/Fe_3_O_4_@PLGA-ZOL NPs, free ICG solution, PLGA NPs, ICG@PLGA and Fe_3_O_4_@PLGA.

The ^1^HNMR spectra were matched with the chemical formula of PLGA-PEG-ZOL and different from the raw materials (PLGA, NH_2_–PEG–NH_2_ and ZOL), the typical nuclear magnetic intensity of hydrogen in PLGA–PEG–ZOL (H1, H2, H3, H4, H5, and H6) showed a corresponding peak (chemical shift δ of H1, 2, 3 = 4.8 ppm, H4 = 2.82 ppm, H5 = 3.67 ppm, H6 = 1.47 ppm) in the ^1^HNMR spectrum of the products, confirming that ZOL was successfully conjugated with PLGA–PEG (Fig. 2a). The average diameter of ICG/Fe_3_O_4_@PLGA-ZOL was first measured to be 313.9 nm with a polydispersity index of 0.03, and there was no obvious change after 24 h, indicating that the dual-targeted NPs had perfect stability in the colloidal solution. The zeta potential of the NPs was − 15.0 mV, which indicated stability in a negatively charged in vivo environment (Fig. 2b,c). The TEM images of the NPs reveal a spherical shape, with nanoscale NPs and many iron particles dispersed in the PLGA shells (Fig. 2d,e). The total ICG amount used in the preparation procedure was 2 mg, while the PLGA amount was 100 mg. The drug loading ratio was 0.227% ± 0.006%, and when the encapsulation efficiency was 8.78% ± 0.38%, the amount of Fe_3_O_4_ was approximately 140 ± 20 µg/ml. The ICG/Fe_3_O_4_@PLGA-ZOL NPs suspended in water were stable, the NPs in the cuvette were magnetically attracted to the side, and the color of the suspension gradually faded after 6 h, confirming the magnetic properties of the NPs (Fig. 2f). The UV–Vis–NIR absorption spectra of the PLGA NPs and Fe_3_O_4_@PLGA NPs show no peak from 450 to 900 nm (Fig. 2g), indicating that PLGA and Fe_3_O_4_ have no light absorption ability. Moreover, the absorption spectra of the ICG/Fe_3_O_4_@PLGA-ZOL NPs show a peak at approximately 800 nm, similar to that for ICG@PLGA NPs and the free ICG solution, while the peak value for the ICG/Fe_3_O_4_@PLGA-ZOL NPs was higher. These results confirm that ICG was successfully loaded in the PLGA NPs and that the absorbance at 800 nm of the ICG/Fe_3_O_4_@PLGA-ZOL NPs is higher than that of the ICG solution, which may have better photo-absorbing ability.

### In vitro PTT effect of ICG/Fe_3_O_4_@PLGA-ZOL NPs

**Figure 3:**
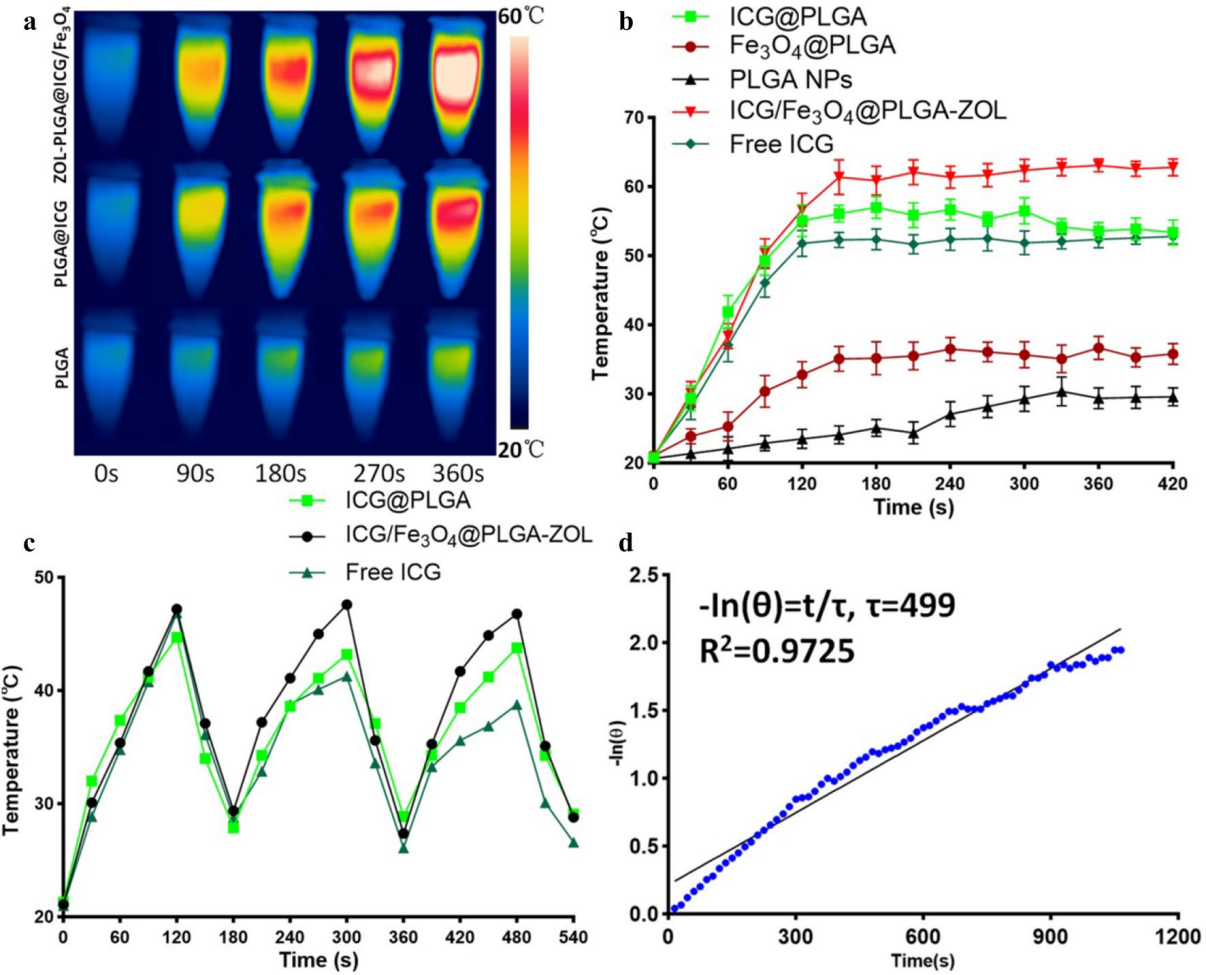
(**a**) Temperatures of various groups measured and imaged by an infrared thermal imaging camera. (**b**) Temperatures of different groups under laser irradiation (808 nm, 1.0 W/cm^2^, 5 min). (**c**) Temperature changes of the ICG/Fe_3_O_4_@PLGA-ZOL NPs, ICG@PLGA NPs and ICG solution in 3 laser ON/OFF cycles. (**d**) Linear regression curve of temperature cooling time (t) vs −ln(θ) of ICG/Fe_3_O_4_@PLGA-ZOL NPs.

To verify the photothermal ability of ICG/Fe_3_O_4_@PLGA-ZOL NPs and other components, an 808 nm NIR laser (1.0 W/cm^2^ for 7 min) was used to irradiate the aqueous suspensions of free ICG, ICG/Fe_3_O_4_@PLGA-ZOL NPs, ICG@PLGA NPs, Fe_3_O_4_@PLGA NPs (with an equivalent ICG concentration of 18 µg/ml) and PLGA NPs in a 1.5 ml EP tube (Fig. 3a). The temperature of the ICG/Fe_3_O_4_@PLGA-ZOL NPs increased rapidly, reaching 63.1 °C, while the highest temperatures of the ICG@PLGA NPs, free ICG, Fe_3_O_4_@PLGA NPs and PLGA NPs were 56.7, 52.8, 36.7 and 30.4 °C, respectively (Fig. 3b). Both ICG and Fe_3_O_4_ may act as light absorbers for PTT, while ICG has a higher photothermal efficiency than that of Fe_3_O_4_. The combination of these compounds could be effective in photothermal conversion, confirming that ICG/Fe_3_O_4_@PLGA-ZOL NPs could be employed as high-efficiency light absorbers for PTT.

To compare the photostability of ICG/Fe_3_O_4_@PLGA-ZOL NPs with that of the free ICG molecules, free ICG, ICG/Fe_3_O_4_@PLGA NPs and ICG@PLGA NPs, the NPs were irradiated in an EP tube for 3 laser ON/OFF cycles (Fig. 3c). The maximum temperatures of the ICG/Fe_3_O_4_@PLGA-ZOL group in 3 cycles were 47.2, 47.6 and 46.8 °C, while those of the ICG@PLGA group were 44.7, 43.2, and 43.8 °C. Both groups showed steady photostability, while ICG/Fe_3_O_4_@PLGA-ZOL had higher efficiency, supporting the conclusion of the in vitro PTT effect. In addition, free ICG also showed great photothermal ability in the first cycle at 46.9 °C but only 41.3 and 38.8 °C in the following cycles. ICG was unstable in aqueous solution, loaded in the PLGA NPs to obtain high photostability, and associated with Fe_3_O_4_ to further enhance the PTT efficiency of the NPs. The photothermal conversion efficiency of ICG/Fe_3_O_4_@PLGA-ZOL NPs was calculated by following equation,

5 $$ \eta = \frac{{hS(T{\max} - Tsur) - Qdis}}{{I(1 - 10^{{ - A_{808} }} )}} $$

6 $$ {\text{h}}S = \frac{msCs}{\tau } $$

7 $$ \theta = \frac{{T - T{\text{sur}}}}{T\max - Tsur} $$

8 $$ t = - \tau In(\theta ) $$

*τ* is determined by a linear regression curve of temperature cooling time (t) vs − ln(θ) of the NPs (*τ*  = 499 s, Fig. 3d). T_max_ is 56.5 °C, T_sur_ is 30 °C, Q_dis_ is measured independently to be 61.7 mW, *I* is 1 W/cm^2^, A_808_ of ICG/Fe_3_O_4_@PLGA-ZOL NPs is 0.91, m_s_ is 1 g, and C_s_ of deionized water as a solvent is 4.2 J/g. Substituting C_s_ into Eqs. (1–4), the photothermal conversion efficiency of ICG/Fe_3_O_4_@PLGA-ZOL NPs is calculated to be 20.77%.

### In vitro cell experiments

**Figure 4:**
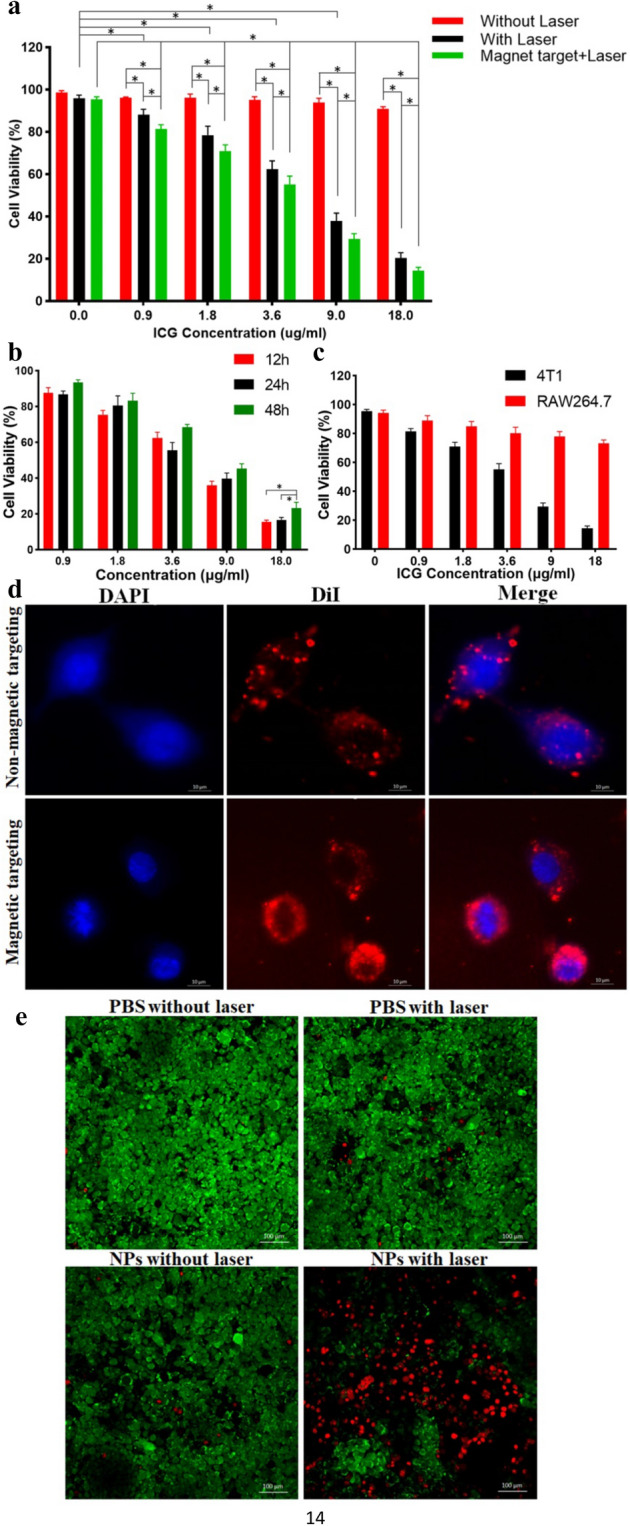
(**a**) Relative cell viability of 4T1 cells in different groups measured by CCK-8 assays at 24 h. (**b**) Cell viability of 4T1 cells co-cultured with ICG/Fe_3_O_4_@PLGA-ZOL NPs with a magnet in the bottom of the plates under NIR irradiation (808 nm, 1 W/cm^2^, 3 min) at 48, 24 and 12 h. (**c**) Cell viability of 4T1 and RAW264.7 cells in group magnet + laser at 24 h. (**d**) Fluorescence images of 4T1 cells incubated with DiI-coated ICG/Fe_3_O_4_@PLGA NPs with or without magnet iron at the bottom of the dish. (**e**) Calcein-AM/PI staining of four groups with different experimental treatments: (I) PBS without laser, (II) PBS with laser, (III) NPs without laser, and (IV) NPs with 3 min NIR laser.

To investigate the cytotoxicity of ICG/Fe_3_O_4_@PLGA-ZOL NPs, and 4T1 and RAW264.7 cells were co-cultured with ICG/Fe_3_O_4_@PLGA-ZOL NPs of different concentrations (ICG concentrations of 18, 9, 3.6, 1.8, 0.9 and 0 µg/ml) in 96-well plates and divided into 3 groups (n = 6): (1) magnet only, (2) laser only, and (3) magnet + laser. Wells without NPs and irradiation served as the controls. The ICG/Fe_3_O_4_@PLGA-ZOL NPs had no obvious cytotoxicity on the RAW264.7 cells even at a maximum ICG concentration of 18 µg/ml at 24 h (Fig. 4a, P > 0.05). Cell viability was 90%, verifying the perfect biocompatibility of ICG/Fe_3_O_4_@PLGA-ZOL NPs. The group without NPs and irradiation showed no significant decrease in cell viability (P > 0.05). The cell viability decreased significantly in the group with NPs under irradiation compared to that of the group with the same NP concentration without NIR irradiation (P < 0.05). Specifically, only 20.34% of the cells were still alive in the group with 18 µg/ml ICG, and 37.87% were alive in the group with 9 µg/ml ICG. The NIR-induced PTT enhanced 4T1 cell ablation. Furthermore, the cell viability of the magnetic targeting group decreased obviously compared to that of the group without a magnet iron on the bottom of the plates (P < 0.05). Considering the group with magnet + laser intervention, more NPs were attracted to the bottom of the dish and were in close contact with the cancer cells, more NPs were engulfed by the cells, and more cells could be killed under irradiation. The CCK-8 results of group magnet + laser at 12 and 48 h showed a similar trend as that at 24 h (Fig. 4b), indicating that the ICG/Fe_3_O_4_@PLGA-ZOL NPs had excellent biocompatibility with cells even after prolonged culture. The groups with magnet and laser irradiation had lower cell viability at 24 h than at 48 h (P < 0.05), and there was no significant difference between 12 and 24 h (P > 0.05), suggesting that the cancer cells regenerated rapidly at 24–48 h after irradiation. The interval between multiple PTT sessions should be within 24 h. The CCK-8 results of RAW264.7 cells showed a similar trend as that at 4T1 cells, ICG/Fe_3_O_4_@PLGA-ZOL NPs had excellent biocompatibility to normal cells, in group magnet + laser, the cell viability of RAW264.7 cells was higher than 4T1 cells, confirmed tumor cells are more susceptible than normal cells to temperature rising (Fig. 4c). The transient exposure to high temperature could significantly inhibit the growth of 4T1 cells, which are due to increased tumor metabolic stresses and the reduced heat-dissipating ability in tumor cell.

The phagocytosis and cell ablation study of ICG/Fe_3_O_4_@PLGA-ZOL NPs was carried out using CLSM to observe directly. DiI (Ex = 549 nm, Em = 565 nm) emitted orange-red fluorescence, and DAPI with an emission wavelength of 400 nm, which was used to stain nuclei, emitted blue fluorescence. The NPs could be attracted to the nuclei after cell phagocytosis, and the red fluorescence intensity was strong in the group with magnetic targeting (Fig. 4d). This result confirmed that magnetic targeting can help gather more NPs to the bottom of the dish and improve the endocytosis of 4T1 cells, which is a critical process in the targeted PTT of ICG/Fe_3_O_4_@PLGA-ZOL NPs. To observe the PTT effect of ICG/Fe_3_O_4_@PLGA NP on cells in culture, the 4T1 cells in dishes were divided into 4 groups: (1) PBS with laser, (2) PBS without laser, (3) ICG/Fe_3_O_4_@PLGA NPs without laser, and (4) ICG/Fe_3_O_4_@PLGA NPs with laser; all cells were stained with Calcein-AM/PI and then observed by CLSM (Fig. 4e). There was no red fluorescence in groups 1, 2 and 3, which had a cell viability of 90%, indicating that neither single laser irradiation nor NPs could ablate the cells, strengthening the safety of ICG/Fe_3_O_4_@PLGA NPs. When the group with ICG/Fe_3_O_4_@PLGA NPs was exposed to the NIR laser, the cell viability obviously decreased to 50%, confirming the excellent PTT effect of ICG/Fe_3_O_4_@PLGA NPs.

### Establishment of an animal model for ultrasound detection

**Figure 5:**
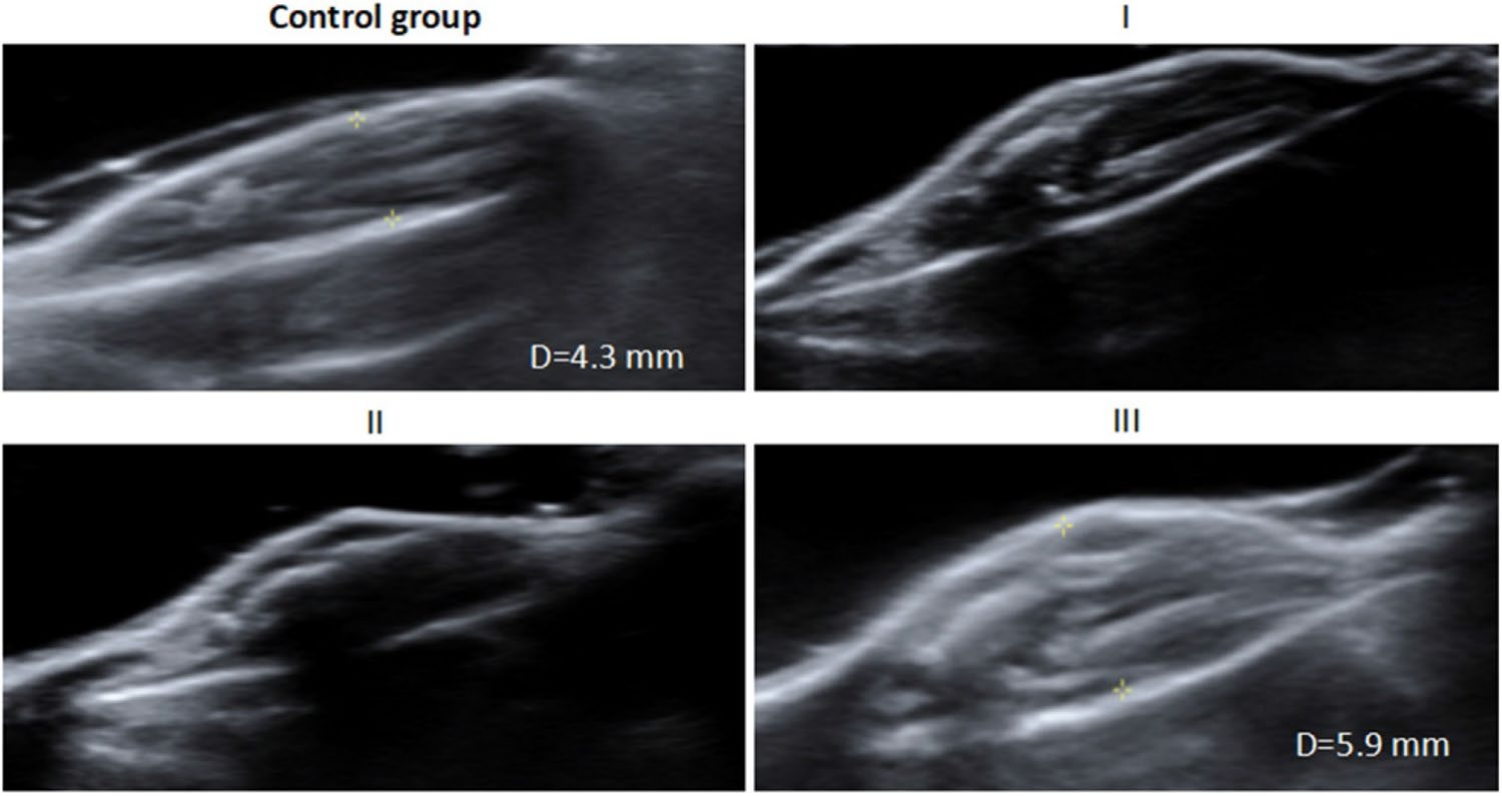
US images of the control and modeling groups on the 5th day after surgery.

To confirm that the mouse BM model was successfully established, US detection was used to inspect the distal femur to proximal tibia of the mice from the third day after surgery (Fig. 5). The US images show that the tibial cortex in the control group was continuous and smooth, the cortical surface of the tibia was firmly attached to the skin, and the diameter of the legs was only 4.3 mm (average diameter of the 20 control legs was 4.67 mm). We imaged the proximal tibia from the third to the sixth day after surgery. The 3 types of US imaging signs in Fig. 4 were mediated to successfully model BM: (1) obvious discontinuity in the proximal tibial cortex, (2) the distance between the cortical surface of the tibia and the skin increased significantly, (3) the diameter of the legs increased by 1 mm compared with that of the control group, and the tibial cortex was rough; the distance of the tibial cortical surface to the skin also increased. This result indicates that cancer cells result in osteolysis of the tibia. These signs appeared in 70% of the mice on the fourth day, and almost all mice on the fifth day were observed to undergo early changes in BM. Therefore, we started to treat mice with BM at an early stage.

### Fluorescence imaging of NPs in vivo

**Figure 6:**
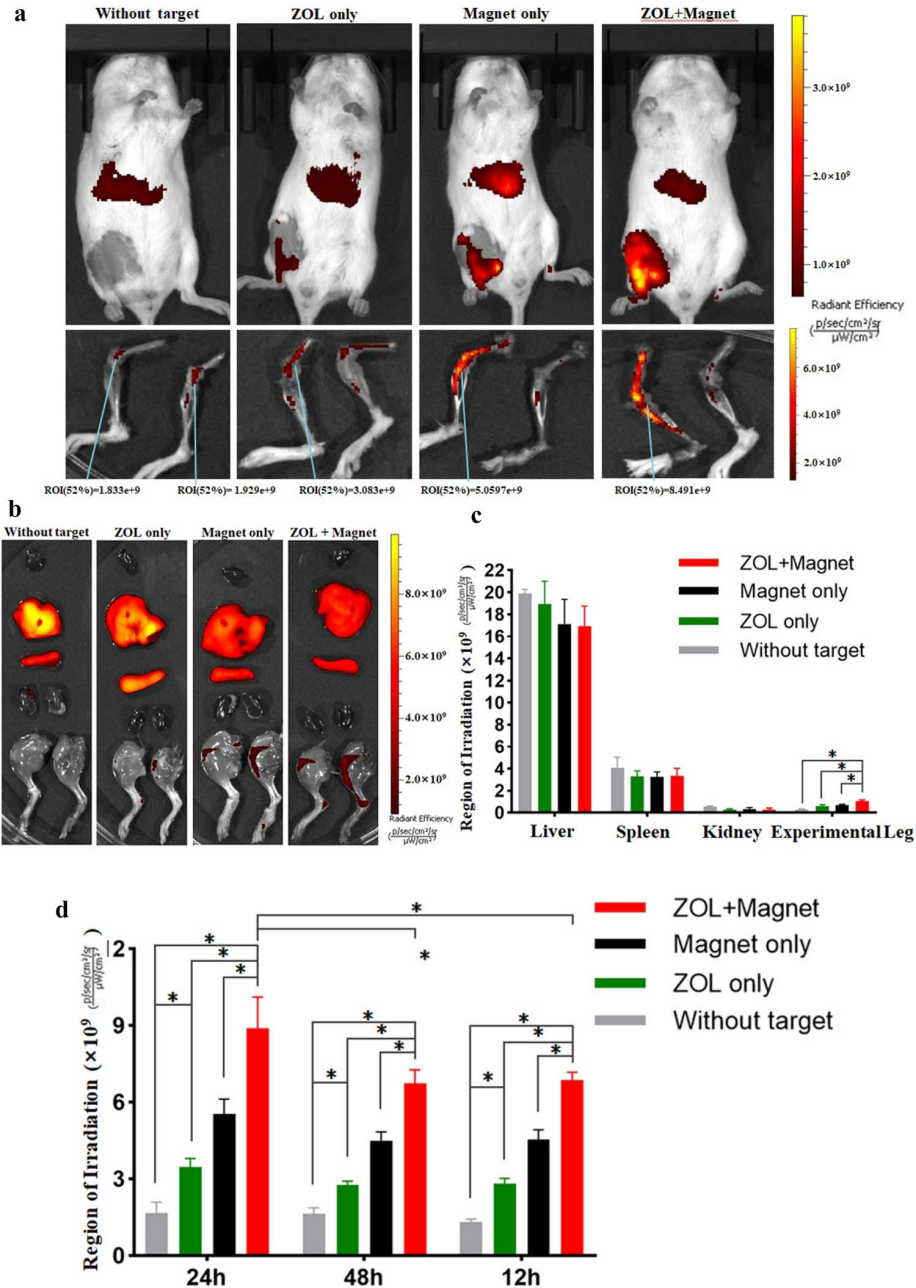
(**a**) IVIS Spectrum imaging system monitoring of NPs in mice with or without targeting at 24 h after intravenous injection. The muscle and soft tissue of the legs were later removed, imaged and measured using the ROI. (**b**) IVIS monitoring of the important organs and both legs harvested from mice. The experimental leg is shown on the right of the picture. (**c**) Average ROI of various organs and experimental legs. (**d**) ROI of skeleton of experimental legs from different groups.

To investigate the in vivo distribution of NPs coated by the target moiety, we fabricated DiR-coated NPs (magnet + ZOL, magnet only, ZOL only and without either), with fluorescent labeling of DiR (Ex = 748 nm, Em = 780 nm). Then, the mice were intravenously injected with the DiR-coated NPs and were all fastened with a circular magnet in the proximal tibia. The IVIS Spectrum imaging system was used to image the mice at 12, 24 and 48 h, and the region of irradiation (ROI) was measured by the corresponding software. The 24 h images (Fig. 6a) show that the fluorescence signal was gathered in the liver of the control group, indicating that most of the NPs aggregated in the liver following blood circulation. The fluorescence signal in the ROI of the experimental leg in the double-targeting group is much stronger than that of the single-targeting group (P < 0.05). The important organ and both legs were harvested and imaged (Fig. 6b). Then, the muscle and soft tissue of the legs were removed, and the remaining skeleton was imaged to observe whether the NPs could reach the medullary cavity of the tibia. There was no fluorescence signal in the heart and almost none in the kidney. Moreover, while strong signals in the liver and spleen were observed, the signals in the leg of the experimental groups were stronger than those in the other groups, representing a high accumulation of NPs (Fig. 6c). The fluorescence signal in the leg of the experimental groups is stronger than in the control group (Fig. 6d), and the single magnet or single ZOL group showed a remarkable bone targeting effect compared with the control group (P < 0.05). Due to the affinity of ZOL to the whole skeleton, the single ZOL group had a slightly increased fluorescence signal in both legs, while the double-targeting NPs enhanced the bone targeting effect compared with the single ZOL or magnet group (P < 0.05). In the magnet + ZOL group, the experimental leg had a significantly stronger signal than that of the offside, and the 12, 24 and 48 h fluorescence images indicate that bone targeting of the magnet + ZOL group at 24 h was highly efficient compared with that of the other groups (P < 0.05), considering that the NPs were still in the process of accumulating at 12 h. Then, the peak concentration in the experimental leg was reached at approximately 24 h, after which the NPs were metabolized; their concentration at 48 h was lower. The results showed that the aggregation of double-targeting NPs can be greatly enhanced in lesions and is better than that of the single-targeting moiety of ZOL or Fe_3_O_4_ (P < 0.05). In vivo, the NPs reached the optimal concentration approximately 24 h after injection, and irradiation at 24 h after injection maximized the effect of PTT.

### In vivo PTT to BM

**Figure 7:**
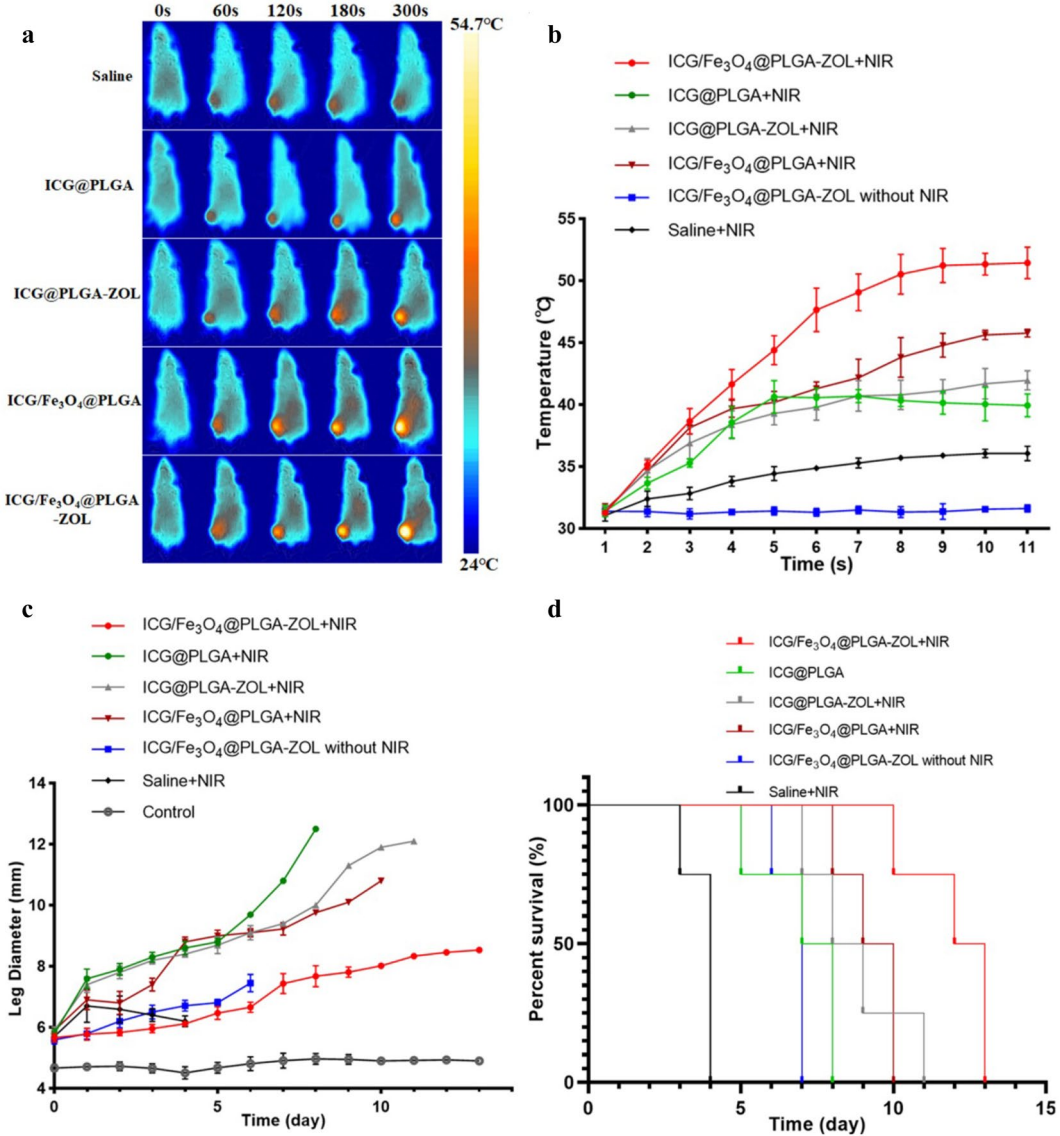
(**a**) Infrared thermal images of mice treated with NPs with NIR irradiation (1 W/cm, 5 min). (**b**) Temperature elevation of the proximal tibia of various irradiated groups. (**c**) Leg diameters of various groups recorded daily. (**d**) Survival curves of mice in all groups (n = 4).

All mice in the animal model were divided into 6 groups (n = 4), (1) saline with NIR laser, (2) ICG/Fe_3_O_4_@PLGA-ZOL NPs without NIR laser, (3) ICG@PLGA NPs with NIR laser, (4) ICG@PLGA-ZOL NPs with NIR laser, (5) ICG/Fe_3_O_4_@PLGA NPs with NIR laser, and (6) ICG/Fe_3_O_4_@PLGA-ZOL NPs with NIR laser. The local temperature of mice under NIR irradiation (1 W/cm^2^, 5 min) was recorded by an infrared thermal camera and showed the photothermal efficiency of ICG/Fe_3_O_4_@PLGA-ZOL NPs (Fig. 7a,b). The local temperature of group 1 increased only to 33.1 °C from 30.7 °C. In contrast, the local temperature of mice injected with NPs with or without the targeting moiety showed an obvious trend of increase. The local temperature of group 6 rapidly increased to 52.4 °C, while that of group 5 increased to 47.9 °C under irradiation. The local temperature of the group injected with ICG@PLGA or ICG@PLGA-ZOL increased to 41.1 and 42.1 °C, respectively. These results indicated that the ICG/Fe_3_O_4_@PLGA-ZOL NPs have a great ability to absorb and transform NIR light into heat and could rapidly increase the local temperature in the irradiated area. Groups 4 and 5, which were injected with a single target moiety, reached higher temperatures under irradiation than those of groups 1 and 3 (P < 0.05). Both ZOL and Fe_3_O_4_ could concentrate NPs at the proximal tibia and obtain a high temperature under irradiation, confirming their targeting properties. Moreover, the temperature of the ICG/Fe_3_O_4_@PLGA group was higher than that of the ICG@PLGA-ZOL group (P < 0.05), suggesting that Fe_3_O_4_ has good targeting efficiency and could endow the NPs with photothermal abilities. The average diameter of the experimental legs on the day of the first treatment was 5.71 mm (Fig. 7c); only one mouse in group 6 remained alive until day 13 and was then euthanized (Fig. 7d).

The leg diameter of group 3 increased to 12.5 mm on day 8, and the mean survival time was 7 days; in group 4, the leg diameter increased to 12.1 mm on day 11 with a mean survival time of 8.75 days; in group 5, the leg diameter increased to 10.80 mm on day 10 with a mean survival time of 9.25 days; however, in group 6, the leg diameter slowly increased to 8.54 mm on day 13 with a mean survival time of 12 days, while the diameter of the contralateral leg in various groups was stable at 4.77 mm. These results indicate that ICG/Fe_3_O_4_@PLGA-ZOL injection with NIR irradiation could effectively prevent the development of BM tumors and prolong the survival time and that the PTT effect of ICG/Fe_3_O_4_@PLGA-ZOL was better than that of ICG/Fe_3_O_4_@PLGA + NIR, ICG@PLGA-ZOL + NIR and ICG@PLGA + NIR. The targeting moiety could aggregate more NPs in the proximal tibia, and the NPs combined with ZOL and Fe_3_O_4_ showed the best therapeutic effect. The ICG/Fe_3_O_4_@PLGA-ZOL without NIR also showed a certain degree of therapeutic effect, which may be associated with the systemic effect of ZOL. In contrast group 1 lived for only 4 days after treatment, and the leg diameter began to decrease after day 2, suggesting that the saline + NIR group rapidly developed into the morbid state, leading to decreases in the weight and bilateral leg diameter after day 2.

**Figure 8:**
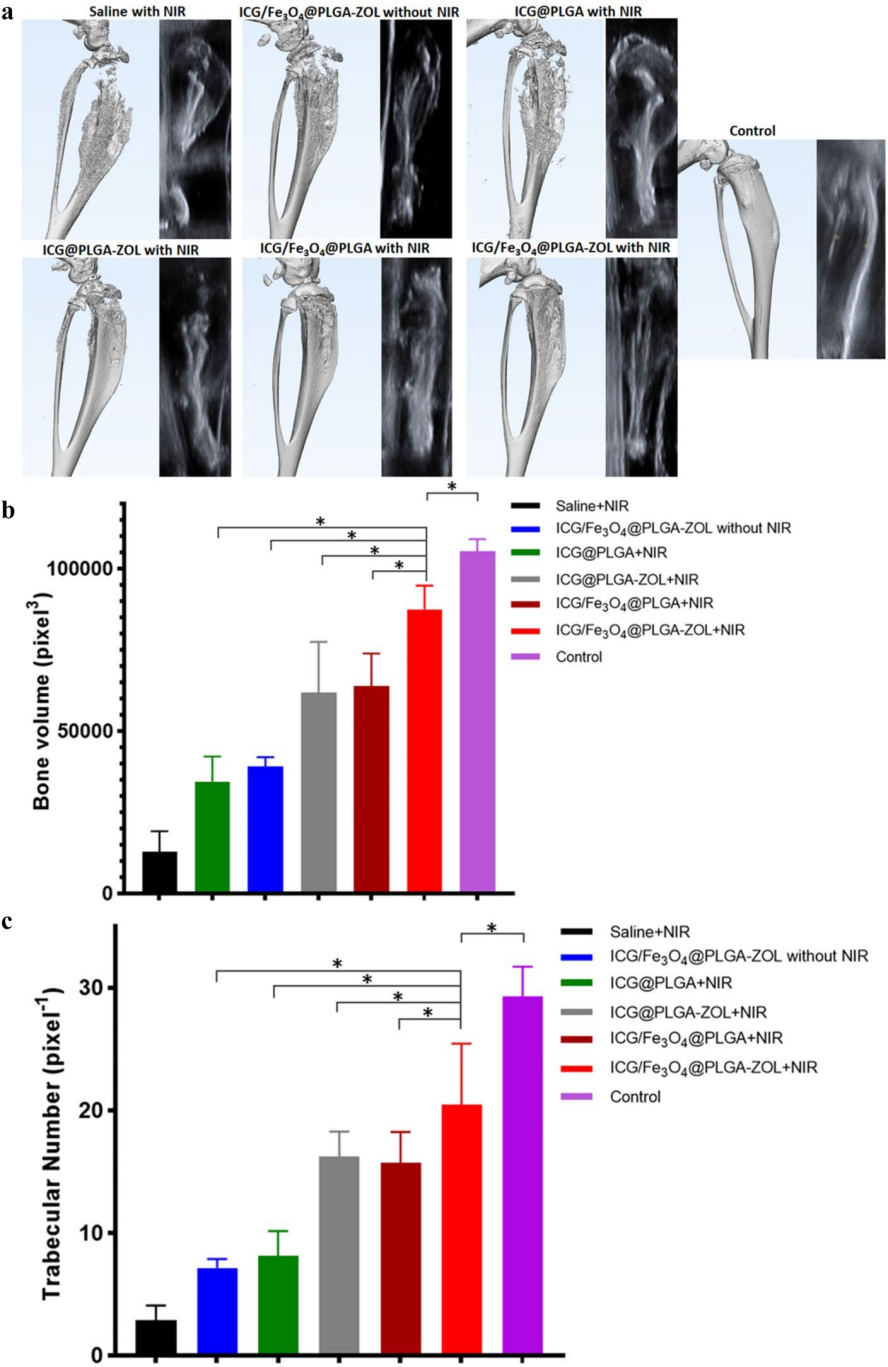
(a) Micro-CT scanning and US detection of legs in all groups: (1) saline with NIR laser, (2) ICG/Fe_3_O_4_@PLGA-ZOL NPs without NIR laser, (3) ICG@PLGA NPs with NIR laser, (4) ICG@PLGA-ZOL NPs with NIR laser, (5) ICG/Fe_3_O_4_@PLGA NPs with NIR laser, and (6) ICG/Fe_3_O_4_@PLGA-ZOL NPs with NIR laser. (**b**) The bone volumes of the sections of proximal tibia in all groups were analyzed by software. (**c**) The trabecular numbers of the sections of proximal tibia in all groups were analyzed by software.

**Figure 9:**
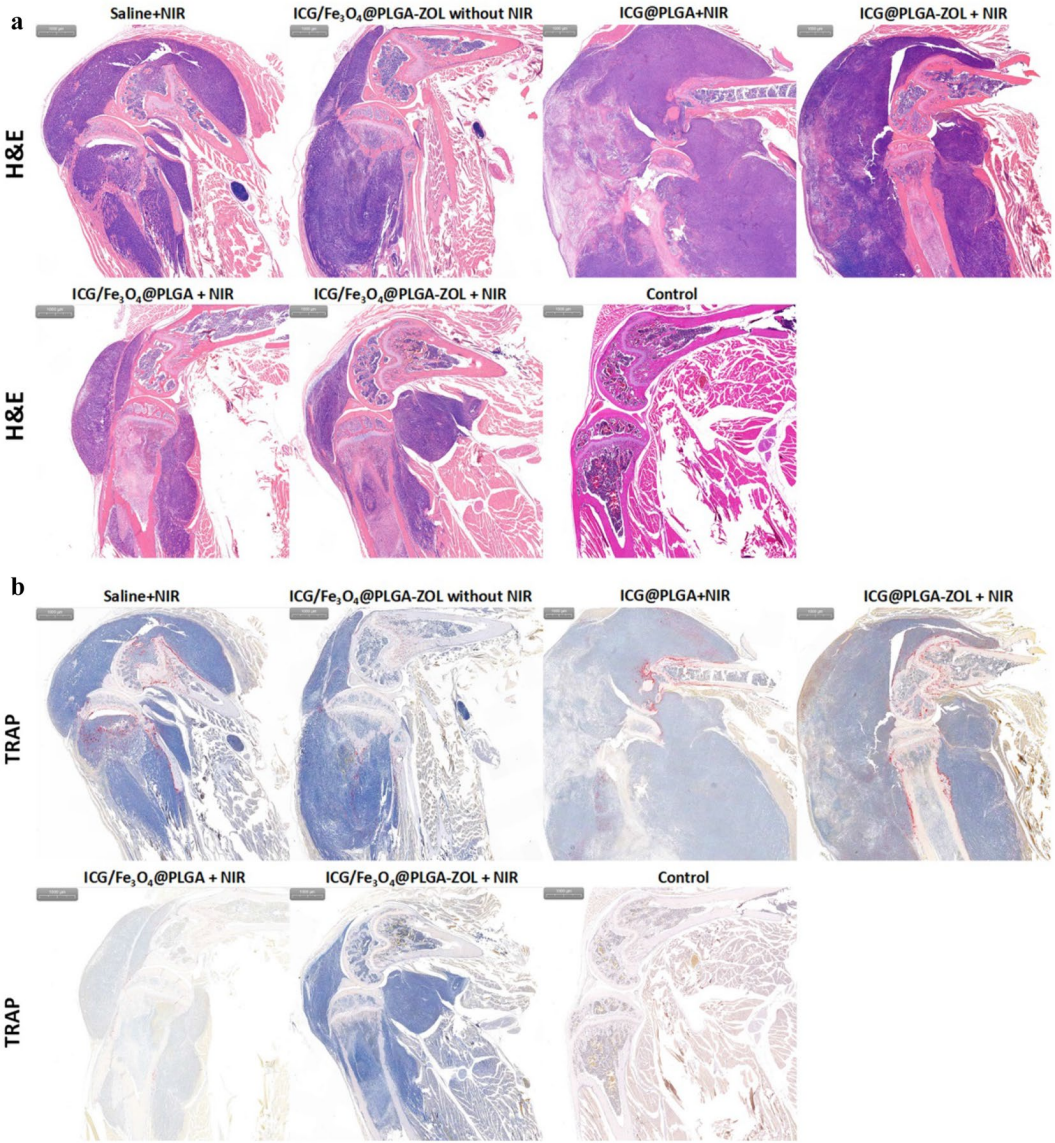
(**a**) H&E staining of experiment leg tissue slices after various treatments (scale bar 1,000 µm). (**b**) TRAP assays of tumor tissue slices after various treatments (scale bar 1,000 µm).

Cancer-associated osteolysis after treatment was observed by micro-CT and US detection (Fig. 8a). Micro-CT scanning revealed that the intratibial cancer-associated osteolysis in group 1 was severe. The bone resorption was obviously diffused from the proximal tibia to the distal tibia and even interrupted the proximal tibia; the average BV and Tb.N (Fig. 8b,c) of group 1 were only 12,962.64 pixel^3^ and 2.91 × 10^–3^ pixel^−1^, respectively, while those of the control group were 105,442.97 pixel^3^ and 29.26 × 10^–3^ pixel^−1^, respectively. The average BV and Tb.N of group 6 were 87,505.72 pixel^3^ and 20.49 pixel^−1^, respectively, which showed a significant increase compared to all those of the other experimental groups (P < 0.05). The CT image of the proximal tibia of group 6 was only slightly rough compared to that of the control group, indicating that the ICG/Fe_3_O_4_@PLGA-ZOL NPs had great PTT efficiency and could attenuate the osteolysis caused by BM. Moreover, the BV and Tb.N of groups 2, 3, 4 and 5 also increased significantly compared to those of group 1 (P < 0.05), and the osteolysis of the proximal tibia was attenuated in the micro-CT images. ICG/Fe_3_O_4_@PLGA-ZOL NPs without NIR laser may attenuate osteolysis through the systemic action of ZOL, and groups 4 and 5 with a single targeting moiety could improve PTT compared to the ICG@PLGA NPs with NIR laser groups. The therapeutic efficiency of groups 4 and 5 was not significantly different (P > 0.05). The US images of groups 1, 2 and 3 show an obvious breakage in the proximal tibia, and the tibial cortex of all experimental groups was rough. The leg diameter and the distance between the tibial tubercle and skin increased significantly, which corresponded to the micro-CT results. Histological assessment was carried out with H&E staining and TUNEL assays to further check the PTT effect on the tumors. H&E staining confirmed that the cancer cells were damaged and that the resorption of bone was controlled upon administration of ICG/Fe_3_O_4_@PLGA-ZOL NPs with NIR laser and targeting (Fig. 9a). In contrast, in groups 4 and 5, the reduction in bone resorption was observed, while single laser irradiation and ICG @PLGA NPs with NIR laser could not suppress bone resorption. Then, TRAP assays were applied to observe osteoclastogenesis in various groups (Fig. 9b). Many TRAP-positive cells with claret staining were found in group 1, but barely any were found in group 6. The degree of bone resorption in both H&E staining and TRAP assays of all groups were completely conform to micro-CT images. Collectively, our data show that the combination of these agents to form ICG/Fe_3_O_4_@PLGA-ZOL NPs and the association with NIR laser irradiation are beneficial for inhibiting the growth of tumor cells and directly preventing bone resorption.

## Conclusion

In conclusion, a nanotheranostic system based on ZOL-modified PLGA NPs with a magnetic targeting moiety and a photothermally triggered drug is fabricated for therapy of BM of breast cancer. The ICG/Fe_3_O_4_@PLGA-ZOL NPs have great biocompatibility, bone-targeting ability and photothermal conversion properties and can inhibit tumor cell growth and bone resorption via the dual actions of bone targeting and PTT. ICG/Fe_3_O_4_@PLGA-ZOL NPs guided by magnetic fields have great potential as cancer theranostics in the near future.
